# Traditional Foods and Starters Utilizing Salt and Osmo-Tolerant Yeast *Hyphopichia burtonii*

**DOI:** 10.3390/microorganisms14081736

**Published:** 2026-08-07

**Authors:** Naganori Ohisa

**Affiliations:** Yamagata Integrated Agricultural Research Center, 6060-27 Minorigaoka, Yamagata 990-2372, Yamagata, Japan; ohisanaga@gmail.com; Fax: +81-22-398-8586

**Keywords:** salt tolerant, *Hypoptychid burtonii*, salami, fermentation starter, Murcha, Daqu, arom

## Abstract

This paper aims to provide an overview of fermented foods involving yeast *Hyphopichia burtonii* and to promote its wider use. The tolerance to salt, osmotic pressure, and heat exhibited by *H. burtonii* is an advantageous trait for survival in the solid state. In traditionally produced salami with a salt concentration of 2.5%, *H. burtonii* became the dominant species after 28 days of ripening and contributed to flavor development. Fermentation starters such as Murcha, Nuruk, and Daqu are produced via salt-free solid fermentation. *H. burtonii* was able to survive the high-temperature conditions associated with solid-state fermentation and drying processes. These fermentation starters used to produce flavorful alcoholic beverages such as rice wine, makgeolli, and baijiu. Certain strains of *H. burtonii* produced aroma compounds such as ethyl caproate and 3-methylthio-1-propanol, contributing to the flavor profile of the beverage. Two strategies can be used to leverage the advantages of *H. burtonii*, such as its salt tolerance and aroma production.

## 1. Introduction

*Hyphopichia*, a type of yeast, is widely distributed in nature and possesses the ability to survive in dry and saline environments. *Hyphopichia burtonii* is the type species of the genus *Hyphopichia* and is a food spoilage microorganism that causes off-odors in fermented bread products [1]. It has also been isolated from diverse substrates including various food sources, freshwater sites and plants, and it is also associated with beetles or beetle larval substrates [2,3]. *H. burtonii* is one of the main yeast species of fermentation starters that possesses amylase used in the production of traditional alcoholic beverages such as Nepal’s “Murcha” [4] and Korea’s “Nuruk” [5]. *H. burtonii* has been found in Japanese dried noodles [6] and white rice preserved for 6 months in closed conditions [7], as well as in Italian ham [8] and the traditional fermented soybean product “giam” [9]. *H. burtonii* is known as chalky yeast that causes white, mold-like defects in par-baked bread [10]. *Saccharomycopsis fibuligera*, *H. burtonii*, *Zygosaccharomyces bailli* and *Saccharomyces cerevisiae* have been reported as dominant species of chalky yeast [11,12,13]. *H. burtonii* is also known as an airborne filamentous fungus that causes food spoilage, alongside *Penicillium nordicum* and *Aspergillus brasiliensis* [14].

**Figure 1:**
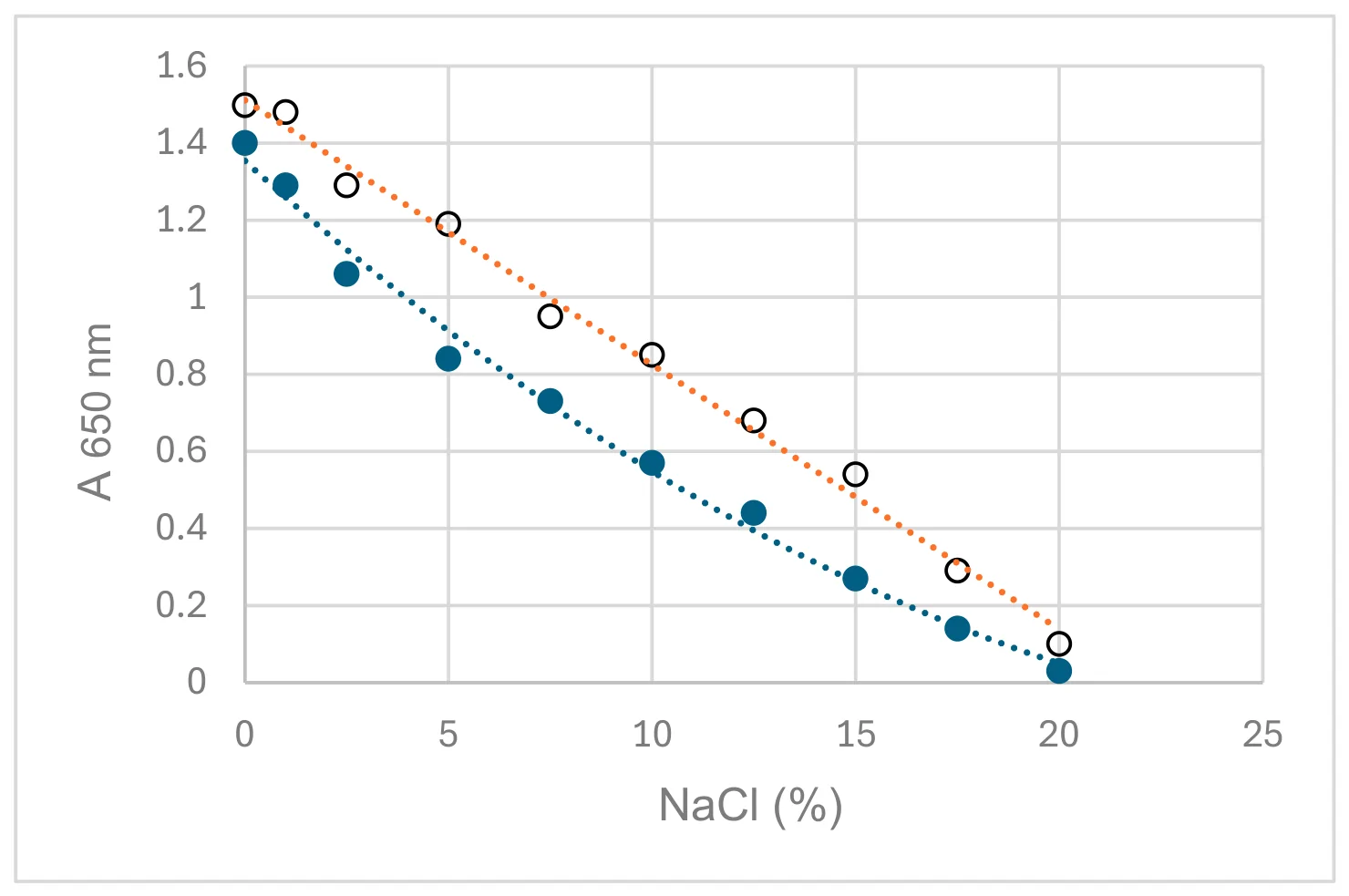
Salt tolerance of *H. burtonii* M1 and M2. M1 (●) and M2 (◯) were cultivated in PD medium at 30 °C for 5 days, Ohisa et al. (2012) [6].

The vegetative cells of *H. burtonii* were oval-shaped, and the spores were cap-shaped [2,6]. Pseudohyphae may be composed of cells of similar size and shape, or they may differentiate into elongated cells and produce blastoconidia [2]. *H. burtonii* can break down starch and then proceed with alcoholic fermentation [4]. Certain strains produced aroma such as ethyl caproate and 3-methylthio-1-propanol [15]. *H. burtonii* was observed to exhibit tolerance to salinity levels ranging from 10% to approximately 20% (Figure 1) [6,15].

Jeong et al. studied the genomic and physiological characteristics of yeast species primarily isolated from Nuruk and Jang [9]. Nuruk and Jang have several prevalent yeast species, including *Saccharomycopsis fibuligera*, *H. burtonii*, and *Debaryomyces hansenii* complex, which belong to the CUG clade showing high osmotic tolerance. The genome of *Hyphopichia* yeasts, is utilized for survival in fermentation environments containing low water activity and high salt concentrations. A group of genes that are induced to be expressed in response to the addition of NaCl and KCl, have been identified in relation to hyperfilamentation growth under high osmotic conditions [16]. When *H. burtonii* exists as pseudohyphae or hyperfilamentation attached to substances such as starch, conventional microbial isolation and purification methods are not very effective. Therefore, the presence and role of *H. burtonii* in fermented foods may be underestimated.

While *H. burtonii* possesses salt and osmotic tolerance, thermotolerance is also a crucial characteristic. The optimum growth temperature of *H. burtonii* strain YHM-G was 25 °C, with a maximum growth temperature of 40 °C [15]. Delgado-Ospina et al. indicated polyphenols could effectively influence thermotolerance of *H. burtonii* isolated from cocoa fermented beans [17]. *H. burtonii* had the capability to accumulate trehalose after a mild heat stress of 46 °C for 2 h, presented during the drying of cocoa fermented beans. In fact, high trehalose accumulation could make the yeast cells resistant to multiple stresses, such as ethanol, heat, and freezing [18]. High concentrations of trehalose stabilize proteins and membranes, thereby enhancing the heat resistance of the cells [19]. Tolerance to salinity, osmotic pressure, and heat is considered to function as an effective mechanism for establishing a unique ecosystem.

*H. burtonii* is a fungus with the unique ability to grow regardless of the presence or absence of salt. It exhibits a dual nature involving both spoilage and fermentation; although classified as a yeast, it behaves like a mold and performs solid-state fermentation. While *H. burtonii* is a fascinating yeast, it has received relatively little research attention compared to other yeasts. Therefore, I intend to gather and organize information on this organism, disseminate the insights gained, and thereby contribute to the advancement of research in this field.

## 2. Fermentation in the Presence of Salt

### 2.1. Japanese Traditional Dry Noodle (Inaniwa Udon)

**Figure 2:**
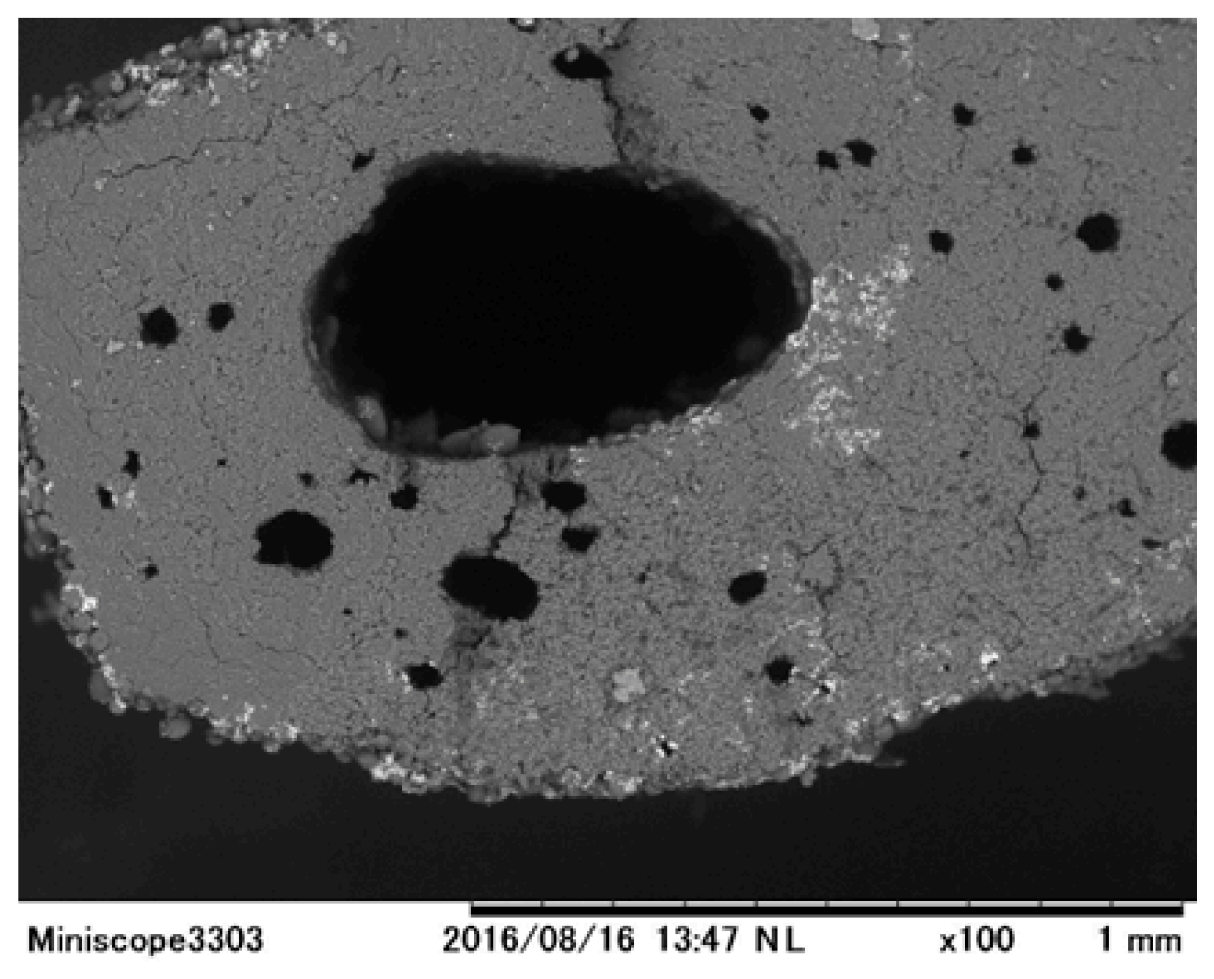
Cross-sectional SEM image of hand-stretched dried noodles (Inaniwa udon).

Inaniwa udon, a type of hand-stretched dried noodle, has a history of nearly 400 years [6]. The dough is made from flour and salt water, flattened, and left to mature overnight. The dough is then cut into thin strips, and rolled into round strings. It has been reported that in hand-stretched noodles, the gluten fibers are stretched in one direction due to the stretching process. Inaniwa udon has many air pockets (voids) that are visible to the naked eye (Figure 2). When dried Inaniwa udon was observed by X-ray CT, it was confirmed that there were voids with a diameter of 10 µm to over 90 µm, and that they were stretched in the rolling direction. Several yeast samples were isolated from the Inaniwa udon dough. These produced gas from sugars and utilized soluble starch. The yeasts were identified as *H. burtonii* by the base sequence of the ITS region and sugar utilization [6].

Machine-made noodles without voids differ in hardness and texture from Inaniwa udon, and that the texture tends to increase as the void ratio increases [20]. However, chewiness tended to decrease when the void ratio exceeded 10%, so it is necessary to control the amount of carbon dioxide generated. The method of controlling the noodles’ shaping characteristics by adjusting the amount of salt added to the dough in winter and summer has been used since ancient times. The manufacturing process involves altering salt concentration, maturation temperature, and maturation time.

Propionic acid is a substance that inhibits the growth of *H. burtonii* and has the potential to serve as a substitute for salt. Among the representative chalk yeasts (*H. burtonii*, *Wickerhamomyces anomalus* and *Saccharomycopsis fibuligera*), *H. burtonii* was the most sensitive to propionic acid [12]. An example of the preservation of *H*. *burtonii* involves the use of sourdough LAB, which naturally produce antimicrobial compounds. The antifungal activity of LAB is primarily attributed to the production of organic acids—such as acetic acid, lactic acid, and phenyllactic acid—as well as cyclic dipeptides [21]. Biopreservation, or the natural preservation of food using microorganisms, is an interesting strategy to replace chemical preservatives such as propionic acid found in bread [21].

### 2.2. Hyphopichia-Enriched Starter for Bread

**Figure 3:**
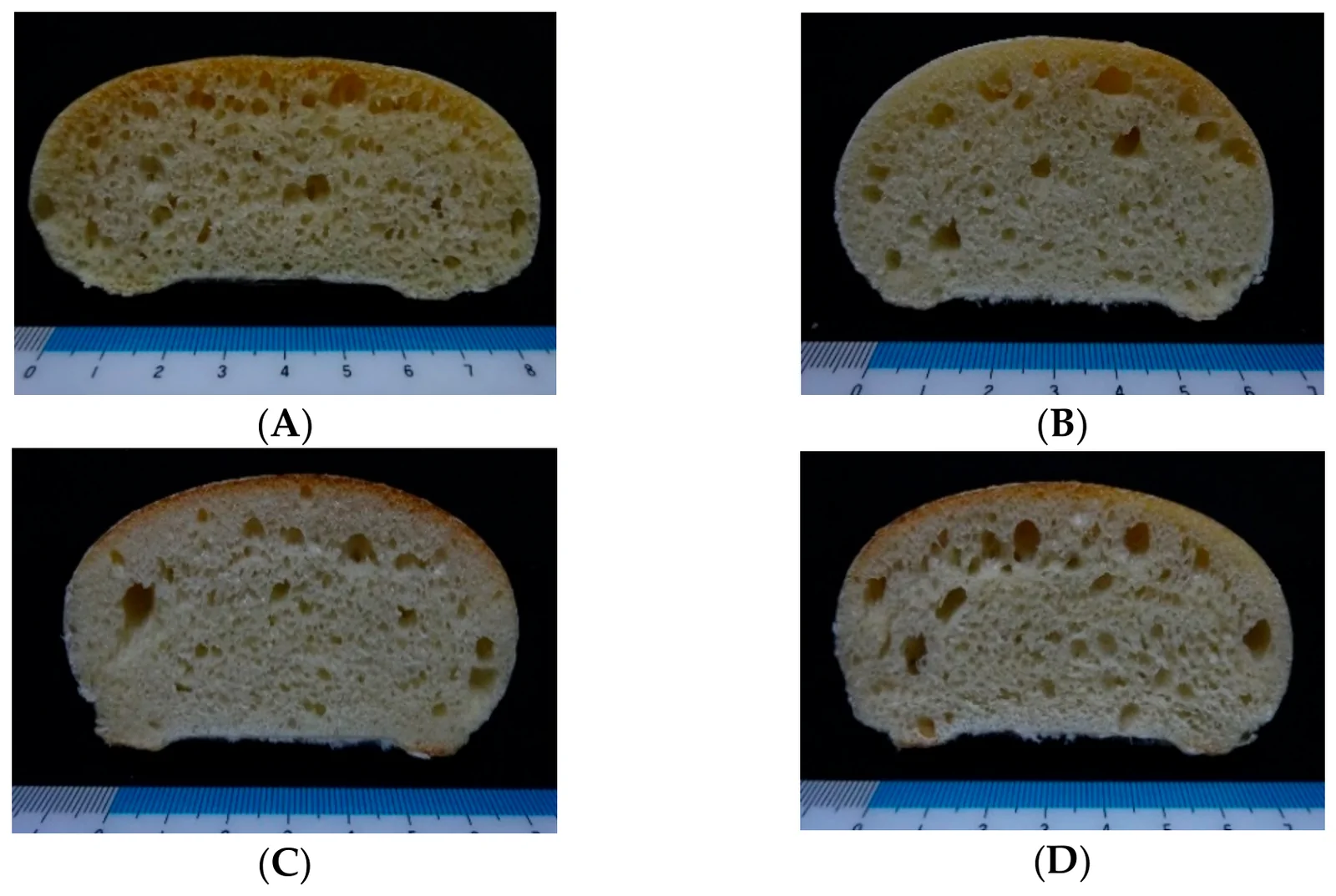
Cross-sectional photograph of bread made from wheat flour with different protein contents. Bread dough with *Hyphopichia*-enriched starters were fermented 24 h at 29 °C. (**A**), weak flour (protein content of 8%); (**B**), medium-strength flour (protein content of 10%); (**C**), strong flour (protein content of 12%); (**D**), strong flour (protein content of 13%). Numerical values in figure indicate centimeter, Ohisa et al. (2023) [28].

Sourdough starter is a paste-like fermentation product obtained by naturally fermenting wheat flour or rye flour [22,23]. In Japan, a type of sourdough starter called Kimoto is made by adding water and rice to koji (rice inoculated with *Aspergillus oryzae*) [24]. Onishi et al. selected *Lactobacillus sakei*, a new cold-growing sourdough variety based on the Kimoto method [25]. The high-throughput sequencing approach has revealed that *H. burtonii* can also be detected in traditional sourdough [26]. To obtain a new starter culture, Inaniwa udon dough was cultured in a 5% saline medium, and the amount of salt-tolerant yeast in-creased to 10^7^ cfu/mL. Unlike conventional sourdough starters, the new starter culture was mainly composed of *H. burtonii* and had a low proportion of lactic acid bacteria (LAB) [27]. The combination of *Hyphopichia*-enriched starter with conventional bread yeast increased the specific volume and softness of the resulting bread. Ordinary baker’s yeast has lost its α-amylase; thus, sugar must be added to the dough to ferment it. However, since *H. burtonii* and the new starter culture possessed amylase activity, wheat flour dough was fermented without the addition of sugar [28]. Furthermore, using only this starter and flour, it was possible to produce bread with a specific volume of 2.0–2.2 cm^3^/g using both strong flour (protein content of 12–13%) and weak flour (protein content of 8%) (Figure 3). *H. burtonii* is thus known as a chalky yeast used to make bread.

### 2.3. Artisanal Salami

Salami sausage is another example in which the salt-tolerant yeast *H. burtonii* is used. Artisanal animal-derived food products are prepared using raw materials of animal origin, sourced from either self-production or specific origins [29]. The production processes for these products are predominantly manual and subject to inspection controls, which aim to preserve the unique, traditional, cultural, and regional characteristics of the product.

Fermented sausages are made using fat, salt, preservatives, seasonings, and ground meat. Typically, a salt concentration of 3–5% is used, but artisanal salami made in southern Brazil uses a lower concentration of 2.5% [30]. In this case, iodized refined salt is added to raw material (meat + fatback). It is known that the salt concentration in fermented meats increases due to dehydration during fermentation [31]. The microbial community in traditional fermented meats influences the product’s quality, color, texture, and flavor [32]. A study identified the microbial community in salami samples throughout the entire ripening process [29]. For fungi, species were identified using second-generation high-throughput sequencing of the intergenic ITS region. Throughout the maturation process, 76 species of fungi from 39 genera were found. The most abundant fungal genera on day 0 were *Yarrowia* (24.96%), *Pichia* (23.91%), *Fusarium* (10.99%), and Candida (10.38%). On day 14, the prominent fungal genera were *Hyphopichia* (73.85%) and *Yarrowia* (18.81%). *H. burtonii* (73.73%), *Aspergillus cibarius* (14.61%), *Wallemia mellicola* (6.30%), and *Yarrowia lipolytica* (4.47%) were predominant on the 28th day of maturation.

Adding specific yeasts as starter cultures to dry-aged meat products enriched the volatile compound profile [33]. Yeasts not only function as a source of flavor but may also act as antagonists against undesirable fungi. High densities of *H. burtonii* have been found even after grease was applied to the surface of ham [34]. Based on their ability to grow on dry-cured ham, twelve yeast strains belonging to the species *Debaryomyces hansenii*, *Debaryomyces maramus*, *Candida famata*, *Candida zeylanoides*, and *H. burtonii* were isolated [35]. These were antagonistic to toxin-producing strains of *Penicillium nordicum* and inhibited ochratoxin A (OTA) biosynthesis. The inhibitory activity of the yeasts decreased with increasing *Penicillium nordicum* inoculation and was enhanced in the presence of NaCl. Furthermore, when high concentrations of *H. burtonii* were present at 30 °C, biological control activity against *Aspergillus niger* and *Penicillium paneum* was also observed [12]. These biological control processes involve microbial communities; *H. burtonii* alone does not act as an antagonist against undesirable fungi.

## 3. Salt-Free Fermentation

### 3.1. Murcha and Nuruk: Amylase and Alcoholic Fermentation

One of the distinguishing features of *H. burtonii* compared to other yeasts is the presence of amylase. *H. burtonii* can break down starch and then proceed with alcoholic fermentation. In the case of sake, saccharification is carried out by *Aspergillus oryzae*, and alcoholic fermentation is carried out by sake yeast.

In oriental countries, people use microbial inoculum made from starchy cereals or grains, either in the form of dry powder or hard balls, to initiate food and alcohol fermentation [36]. These starters are known by different names across the region, such as Murcha or Marcha in the Himalayan regions of Nepal, India, and Bhutan; Ragi in Indonesia; Chinese yeast in Taiwan; and Nuruk in Korea [37,38]. Marcha, specifically, is a mixed dough inoculate employed as a starter culture to produce various traditional alcoholic beverages like jaanr and raksi in Nepal, India, and Bhutan.

Karmacharya et al. collected Marcha samples from 10 different geographic regions of Nepal, obtaining 27 samples altogether [36]. The isolates were separated into Groups A, B, and C based on morphological and physiological characteristics. Group B yeast displayed high amylase production and the ability to produce ethanol. The molds were thought to be major starch degraders in the Marcha, but it was assumed that two types of yeast are involved in fermentation: amylolytic yeasts which hydrolyze starch, and alcohol-producing yeasts which convert hydrolyzed starch to ethanol [39]. Among more than 20 yeast strains isolated from the traditional starter Murcha, Takeuchi et al. characterized a yeast that might be involved in saccharification [4]. This strain was identified as *Pichia burtonii* (*H. butonii*), and when cultured in the presence of starch in the medium, it produced extracellular α-amylase. *H. butonii* isolated from most of the Murcha from Nepal suggested that this strain plays a significant role in saccharification and might contribute to flavor formation in the beverage [4].

Ragi (Indonesia) and Men (Vietnam) are examples of amylase starters used to produce rice wine [40]. These starters are primarily made by adding a mixture of local herbs and spices to uncooked rice flour. The flour and herbs are moistened with a small amount of water to make a dough, which is then formed into small balls and spread on a culture tray, over which pre-powdered Ragi or Men is sprinkled [41]. The microbial flora of Ragi or Men can grow in the fresh dough overnight at high humidity and a temperature of about 30 °C. The next step is to carefully dehydrate the microbial starter to stabilize and preserve it. This is achieved by drying it for several days in a room artificially heated to about 60 °C.

Makgeolli, a traditional Korean fermented rice wine, is made using a traditional fermentation starter called Nuruk [9]. Nuruk is made by natural fermenting coarsely ground grains with water, after which the dough is dried for 2–3 weeks to become Nuruk cake. Next, the Nuruk cake is added to cooked rice and fermented in a liquid state (mash). Makgeolli is made by filtering the mash, while traditional distilled spirits are made by distilling the mash. Murcha, Nuruk, Ragi, and Meju involve solid-state fermentation, and *H. burtonii* have been isolated as fungal species. Drying process reduces the water activity (Aw) of Murcha, Nuruk, and Ragi, creating an environment suitable for the survival of *H. burtonii*. This suggests that *H. burtonii* was able to withstand a drying temperature due to its osmo and thermotolerance [17].

Table 1 summarizes several foods containing yeast and fungi, along with the amount of salt added. Yeast has limited tolerance to NaCl, and at a concentration of 15%, the growth of most microorganisms is inhibited. However, *H. burtonii* can grow with or without the addition of salt (Figure 1). In Murcha, Nuruk, and Meju, water activity (Aw) decreases during the drying process after fermentation, but *H. burtonii* counteracts this through its osmotic tolerance. *H. burtonii* and *H. pseudoburtonii* possessed gene families of amino acid permease and ATP-binding cassette transporters, whose dynamic expressions patterns during osmotic stress were revealed [19]. Furthermore, the increase in glycerol accumulation during osmotic stress is mediated by efficient production via cytoplasmic Gpd1p. *Zygosaccharomyce rouxii*, which grows in soy sauce and miso, and can also grow in environments with a salt concentration of approximately 20%. *Zygosaccharomyce rouxii* possesses mechanisms that alter the lipid composition of the cell membrane and Na+-ATPase that expels sodium from the cell [42]. It can also regulate osmotic pressure through the accumulation of glycerin. Treatment of *Zygosaccharomyce rouxii* with high concentrations of salt has been shown to increase the concentrations of volatile substances such as 3-methyl-1-butanol and phenylethyl alcohol [43].

### 3.2. Aromatic Compounds Producer

While it has been shown that certain strains of *H. burtonii* produce styrene, causing an unpleasant odor in fermenting bread products [1], other strains exist that produce aromatic compounds. According to research by Morozumi et al., styrene was produced from cinnamic acid added to boiled beans in conjunction with the growth of *Candida famata* and *Pichia membranaefaciens* [53]. It has already been established that *Saccharomyces cerevisiae* possesses the ability to produce styrene [54]. Similarly, it is believed that in the case of *H. burtonii*, a styrene odor does not develop unless cinnamic acid is present.

Korean distilled spirits are made by distilling Nuruk mash. Small-scale brewing tests of Nuruk, made from rice flour, revealed that it contains a high concentration of ester compounds such as ethyl isobutyrate, ethyl α-methyl butyrate, ethyl isovalerate, ethyl heptoate, and ethyl nonaoate [55]. Traditional Chinese baijiu has been widely consumed for thousands of years and is renowned overseas [56,57]. Traditional baijiu is normally made using solid-state fermentation with sorghum or a mixture of grains [44,45]. The raw materials, either whole grains or powdered grains, are mixed and then steamed. The fermentation starter, Daqu, is added to the steamed mixture, and fermentation is carried out for one to two months. Finally, the fermented mixture is distilled in its solid state to produce fresh baijiu. Daqu (18–20% of the raw materials) is mixed in for saccharification and fermentation and serves as an important source of microorganisms in the fermentation process of baijiu. These microorganisms also influence the taste of baijiu.

Daqu is generally produced as follows: The barley and peas are combined and ground, water is added (41–44%), and the mixture is compressed into a flat block (22.5 × 17.5 × 8.5 cm) [46,47]. Blocks are stacked in a fermentation room and fermented for about a month. In Fengxiang-type baijiu, the core temperature reaches 58–60 °C and is maintained for three days. This Daqu has a high moisture content and acidity, and contains many bacteria, so it should be dried and stored for three months before use. Although storage reduces the number of LAB, yeast content, and enzyme activity, using stored Daqu results in a relatively slower fermentation temperature during brewing, enhances the flavor of the baijiu, and increases the baijiu recovery rate.

Li et al. analyzed the microbial community dynamics of Daqu and isolated three high-yield ethyl caproate-producing yeasts (Saccharomyces cerevisiae Y7#09, *H. burtonii* F12507, and *Clavispora lusitaniae* YX3307) [48]. The microbial community was investigated using Illumina HiSeq technology. Forty-one flavor compounds were detected in all samples, including seven alcohols, 26 esters, and four aldehydes. An increase in the content of ethyl caproate, which has an apple-like aroma, was associated with Daqu fortified with the mixed inoculum. The ester content of fortified Daqu was higher in the later stage of fermentation than that in unfortified Daqu.

It was thought that the ethyl caproate contained in the sludge was synthesized as follows: *Clostridium caproicum*, which inhabits the sludge, produces caproic acid during the fermentation process, which is then esterified with ethanol by enzymes in the solid mash [46,47]. Fatty acid ethyl esters are secondary metabolites produced by *Saccharomyces cerevisiae* and many other fungi. In *Saccharomyces cerevisiae*, ethyl caproate is produced by the coupling of acetyl-CoA caproic acid with ethanol [58]. This coupling reaction is carried out by the genes Eeb1p and Eht1p, which encode acyl-CoA:ethanol O-acyltransferase (AEATases). The same appears to be true for *H. burtonii* F12507 isolated from Daqu. It is necessary to determine whether the Eeb1p and Eht1p genes are present in the genome of *H. burtonii*. This is because *H. burtonii* Tip11, recovered from zebra dove droppings, has a genome length of 12,360,159 bp and a GC content of 35.16%, and encodes 2146 proteins closely related to those of the *Saccharomycetaceae* family [49].

Xiaoqu is also a traditional Chinese fermentation starter that uses rice as its raw material [50]. The name Xiaoqu comes from its smaller size compared to Daqu, and its production method is similar to that of Korean Nuruk or Japanese koji. Wang et al. investigated the fungal diversity of six Xiaoqu samples from China and screened for low-alcohol fermenting yeasts [51,59]. Fifteen fungal species were detected, and similarities in fungal diversity were observed among the Xiaoqu samples. The fifteen fungal species consisted of 11 filamentous fungi, including the most common species *Rhizopus arrhizus*, and four yeast species, including *H. burtonii*, *Saccharomyces cerevisiae*, *Saccharomycopsis fibuligera*, and *Saccharomycopsis malanga*.

3-methylthio-1-propanol (3-Met), a sulfur-containing higher alcohol, is an important aroma component in many foods and beverages, including soy sauce, cheese, baijiu, beer, and wine [60]. It is also naturally found in foods such as apples and tomatoes and has a low odor threshold of 1–3 ppm [52]. One strain, YHM-G, which produced a high level of 3-Met, was isolated from the baijiu-producing environment and identified as *H. burtonii* according to its morphological properties, physiological and biochemical characteristics, and ribosomal large sub-unit 26S rRNA gene D1/D2 domain sequence analysis [15].

In China, baijiu with a distinctive aroma is highly favored, and it is classified into different types (xiāngxíng) based on its aroma and taste [44]. The key flavor compounds of nongxiangxing baijiu were ethyl caproate, p-cresol, and γ-nonolactone. Nonanal, ethyl decanoate, and phenylethanol were the characteristic flavor compounds in qingxiangxing baijiu. Ethyl caproate, ethyl caprylate, and isoamyl acetate are known to be important components that affect the quality of Japanese sake [61].

## 4. Risks Associated with *Hyphopichia burtonii*

*H. burtonii* is known to cause skin infections in the Western barbastelle bat (*Barbastella barbastellus*) and the greater bulldog bat (*Noctilio leporinus*) [62,63]. Until recently, there were no reported cases of infection in humans. In 2021, a case was reported involving a patient undergoing peritoneal dialysis who was initially diagnosed with sterile peritonitis but was actually suffering from peritonitis caused by *H. burtonii* [64]. This pathogen posed a challenging diagnosis, causing low-grade peritonitis and difficulty to culture with standard bacterial broth. Broad-range PCR targeting rDNA followed by DNA sequencing successfully solved the etiology. Mayra et al. reported the case of a 44-year-old woman with end-stage renal disease who developed peritonitis while undergoing continuous ambulatory peritoneal dialysis [65]. Feldman et al. isolated *H. burtonii* from the peritoneal fluid of patients with liver cirrhosis and secondary peritonitis [66]. In the initial human case report, *H. burtonii* was susceptible to amphotericin B, voriconazole, fluconazole, itraconazole, and caspofungin [65].

*H. burtonii* is known as an airborne, filamentous fungus that causes food spoilage. Berni et al. evaluated the resistance of two pathogenic bacteria and three airbome food-spoiling fungi (*H. burtonii*, *Penicillium nordicum*, and *Aspergillus brasiliensis* ATCC 16404) to gaseous ozone on stainless steel [13]. Ozone exposure at 4–12 mg/L was effective against pathogenic bacteria; however, for filamentous fungi, a significant effect was observed only at a concentration of 50 mg/L against a monolayer inoculum.

Air acts as a vehicle for the dispersal of fungal propagules in food processing environments, serving as a source of cross-contamination between products. Selecting a disinfectant with sufficient antifungal activity is a critical point for minimizing losses associated with fungal spoilage in the food industry [67]. Six isolated strains known to cause spoilage in bread products (*Penicillium roqueforti*, *Penicillium paneum*, *H. burtonii*, and *Aspergillus pseudoglaucus*) were tested against five types of sanitizers—benzalkonium chloride (0.3%, 2.5%, 5%), biguanide (2%, 3.5%, 5%), peracetic acid (0.15%, 1.5%, 3%), quaternary ammonium compounds (0.3%, 2.5%, 5%), and sodium hypochlorite (0.01%, 0.1%, 0.2%) [68]. Peracetic acid proved to be the most effective sanitizer across the genera, species, and concentrations evaluated, generally achieving a 2- to 4-log reduction in the initial microbial load.

To address the adverse effects of *H. burtonii*, monitoring is required in addition to factory and environmental sanitation. One common method involves using PD agar medium supplemented with chloramphenicol [69]. This method, which captures airborne microorganisms by exposing PDA plates to the air for a set period, is suitable for small-scale factories. A microbial counter is a type of flow cytometer that detects light scattered by particles when a sample is irradiated with a laser beam [70]. By simultaneously detecting fluorescence alongside scattered light, it is possible to distinguish between microorganisms and non-biological particles. Since this method allows for continuous, real-time measurement, it is an effective approach for risk management. Microbial community analysis using high-throughput sequencers is a method that involves extracting DNA without isolating individual microorganisms, performing PCR amplification with universal primers, and comprehensively analyzing the resulting nucleotide sequences. This article demonstrates that the method enables the rapid, simultaneous assessment of a wide variety of microorganisms within a microbial community [9,15,26,30]. In fermentations where *H. burtonii* plays a dominant role, it cannot be excluded from the process. Isolating fermentation vessels, maintaining factory sanitation, and conducting environmental monitoring are necessary to control airborne contamination.

## 5. Conclusions

**Figure 4:**
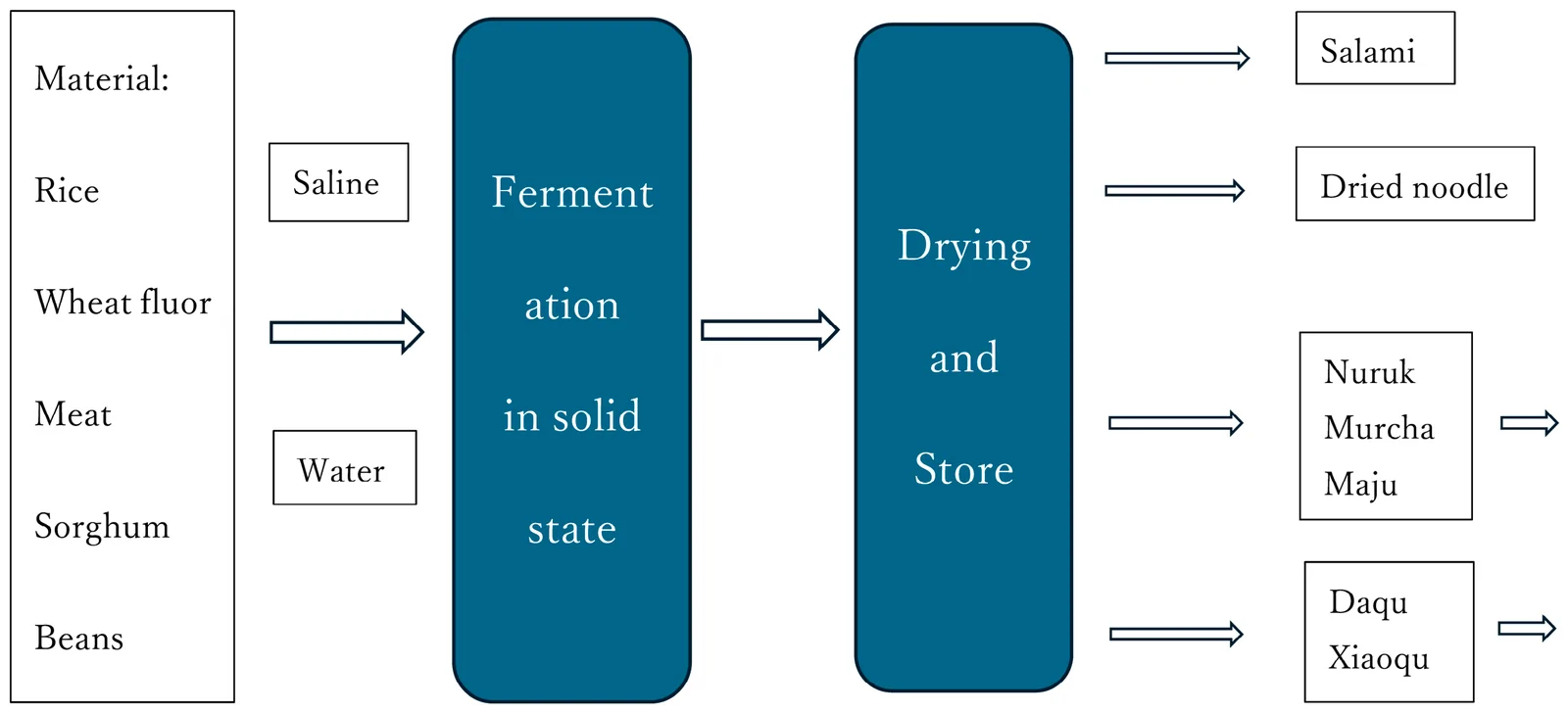
Several starter cultures and fermented products involving *H. bartonii*. Fermentation can be divided into methods using saline and methods that do not. Fermented starters (such as Nuruk, Murcha, Daqu, etc.) are produced by solid-state fermentation and dry at 40 °C.

Figure 4 shows several starter cultures and fermented products including *H. burtonii*. A type of handmade dried noodle, Inaniwa udon, is made from wheat flour and 5% saline. The salt tolerant yeast *H. burtonii* ferments within the dough and creating cavities. Artisan salami found in southern Brazil uses a low salt concentration of 2.5%. After 28 days of maturation in dry conditions, *H. burtonii* (73.73%) became dominant. Fermented starters such as Nuruk, Ragi, Meju, and Daqu are produced using solid-state fermentation, dried, and then used as starter cultures. This diagram shows two processes: fermentation with or without salt, and drying prosses at 40 °C or room temperature.

As seen in salami and dried noodles, brine fermentation is favorable for the growth of *H. burtonii*. Because *H. burtonii* possesses gene families of amino acid permeases and ATP-binding cassette transporters, making it effective against salt and osmotic stress. In the case of saltless fermentation (Nuruk, Murcha, Meju, Daqu), drying at 40 °C and storing reduces contaminating microorganisms. As *H. burtonii* was heat-tolerant, it is considered suitable for drying at 40 °C and for high-temperature solid-state fermentation processes such as Daqu.

Two strategies can be considered to take advantage of the characteristics of *H. burtonii*, such as its high osmotic tolerance and fragrance production. One approach involves adding 2–3% salt during solid fermentation. This is favorable for the growth of *H. burtonii*. Another approach is to increase the use of fermented starters to other fermented foods. Nuruk could potentially be used as a starter culture in the production of low-sodium miso, low-sodium soy sauce, and pickles. Using traditional Chinese brewing starters makes it easy to select highly aromatic fermentation yeasts. Adding *H. bartonii* strainYHM-G, which produces 3-Met, during the later stages of fermentation may result in sake with a distinctive aroma. Section 4 provides useful information regarding the safety of this strain and factory management. It is also necessary to regularly quantify 3-Met to manage the quality of the sake.

## 6. Future Research Prospects

Intriguing questions remain regarding the aroma. How does the aroma-producing *H. burtonii* proliferate, and what conditions are required for aroma generation? Aroma production may be linked to excessive filamentous growth (hyperfilamentation) under high-osmotic-pressure conditions; indeed, it has been shown that treating *Zygosaccharomyces rouxii* with high salt concentrations leads to increased levels of volatile compounds such as 3-methyl-1-butanol and phenylethyl alcohol [43]. Furthermore, aroma production might be associated with extreme temperature conditions or semi-anaerobic environments. There is also a need to elucidate the biosynthetic pathway of ethyl caproate in *H. burtonii* and determine whether it is a species-specific product or a characteristic shared across the *Hyphopichia* genus.

## Figures and Tables

**Table 1:** Fermented foods involving fungi and yeast, and the amount of salt added.

Foods	NaCl Addition (%)	Related Fungi and Yeast	Reference
Dried Inaniwa noodle	4–7	*H*. *burtonii*	[6,20]
Artisanal salami	2.5	*H*. *burtonii*, *Aspergillus*, *Wallemia*, *Yarrowia*	[30,35]
White rice (stoked at 5 °C)	No addition	*H*. *burtonii*, *Aspergillus tritici*	[7]
Bread	1–2	*Saccharomyces cervicis*, *H*. *burtonii* *	[27,28]
Murcha	No addition	*Mucor circinelloides*, *Rhizopus chinensis*, *Saccharomycopsis fibuligera*, *Pichia anomala*, *H*. *burtonii*, *Saccharomyces cerevisiae*	[4,36,37,38]
Nuruk	No addition	*Saccharomycopsis fibuligera*, *H*. *burtonii* *Aspergillus*, *lichtheimia*, *Mucor* spp.	[9]
Meju	No addition	Airborne mold, *H*. *burtonii*	[9]
Jang	9–20	*Saccharomycopsis fibuligera*, *H*. *burtonii*, *Debaryomyces hansenii*	[9]
Daqu	No addition	*Saccharomycopsis fibuligera*, *H*. *burtonii* *Pichia kudriavzevii*, *Rhizopus oryzae*, *Sterigmatomyces elvai*, *Aspergillus flavus/oryzae*	[15,44,45,46,47,48]
Xiaoqu	No addition	*Rhizopus arrhizus*, *H*. *burtonii*, *Saccharomyces cerevisiae*, *Saccharomycopsis fibuligera*, *Saccharomycopsis malanga*	[49,50,51]
Saka-tane (Tane koji)	No addition	*Aspergillus oryzae*, *Aspergillus usamii*	[24]
Japanese sake	No addition	*Aspergillus oryzae*, *Saccharomyces sake*, *Hansenula anomala*	[52]
Miso	5–13	*Zygosaccharomyce rouxii*	[42]
Soy sauce	16–19	*Zygosaccharomyce rouxii*	[42]

* Chalk molds.

## Data Availability

No new data were created or analyzed in this study. Data sharing is not applicable to this article.

## Abbreviations

The following abbreviations are used in this manuscript:

LAB	Lactic acid bacteria
3-Met	3-Methythio-1-propanol
PD-medium	Potato dextrose medium
*H. burtonii*	*Hyphopichia burtonii*
